# Identical twins:one with anti-glomerular basement membrane glomerulonephritis,the other with systemic lupus erythematosus

**DOI:** 10.1186/1471-2369-14-277

**Published:** 2013-12-20

**Authors:** Xiaoxia Liu, Yu Wu, Yuan Yang, Jue Wang, Ye Tao, Ping Fu, Zhangxue Hu

**Affiliations:** 1 Department of Nephrology, Chengdu, Sichuan Province, China; 2 Department of Hematology and Hematology Research Laboratory, Chengdu, Sichuan Province, China; 3 Department of Medical Genetics-all in National Key Laboratory of Biotherapy of Human Diseases, West China Hospital, Sichuan University, Chengdu, Sichuan Province, China; 4 Institute of Blood Transfusion, Chinese Academy of Medical Science, Chengdu, Sichuan Province, China

**Keywords:** Anti-GBM nephritis, HLA-DRB1*1501, Systemic lupus erythematosus, Identical twins

## Abstract

**Background:**

Anti-glomerular basement membrane (GBM) glomerulonephritis and systemic lupus erythematosus (SLE) are both disorders of the immune system; however, they are known as distinct diseases. Till now no clinical evidence suggests the genetic relationship between these two diseases. Herein, we present two identical twins; one was diagnosed as anti-GBM glomerulonephritis, the other SLE. This is the first clinical report on the genetic relationship between these two diseases.

**Case presentation:**

A 25-year-old female was admitted complaining of intermittent gross hematuria for 6 months and elevated serum creatinine for 1 month. She denied hemoptysis. Laboratory examinations showed hemoglobin 7.4 g/dL, serum creatinine 7.15 mg/dL and albumin 2.8 g/dL. Urinalysis showed hematuria (484 RBCs per high-power field) and proteinuria 4+. Antinuclear antibody, complement levels and ANCAs were all normal. Renal ultrasound showed normal-sized kidneys without obstruction or masses. Serum anti-GBM antibody assay showed 119.70 RU/mL (normal range, <20 RU/mL). Chest X-ray was normal. She was diagnosed as anti-GBM glomerulonephritis and received plasma exchange (2000-3000 ml plasma/exchange, 5 turns), methylprednisolone 0.5 g for three days, plus cyclophosphamide. Although serum anti-GBM antibodies decreased gradually to a normal range, her renal function did not improve. One month later, her identical twin sister was diagnosed as SLE based on malar erythema, arthralgia, antinuclear antibody positive with liter 1:1000, and Anti-Smith (Sm) antibody ++. Anti-GBM antibody and complements were within normal ranges. Further study showed these twins were HLA-DRB1*1501 homozygotes.

**Conclusion:**

The presence of identical twins having anti-GBM nephritis and SLE respectively provides clinical evidence to support that anti-GBM nephritis and lupus may share a common genetic background to some extent, while environment may contribute to disease evolution in part.

## Background

Lupus is a prototypic autoimmune disease and pathogenesis involves genetic predisposition and environmental conditions. Its highest reported concordance rate in monozygotic twins is up to 57% [1]. Anti-GBM nephritis is also an autoimmune disease, but is much more rare and with a lower incidence compared to lupus. This disease can also occur in siblings and sets of identical twins [2]. However, there is no reported case of identical twins suffering from anti-GBM nephritis and lupus separately. We report a 25-year-old female developing anti-GBM nephritis, and her identical twin sister developing systemic lupus erythymatosus (SLE). Their HLA genotypes were both homozygous for HLA-DRB1*1501.

## Case presentation

A 25-year-old female was admitted to our hospital complaining of intermittent gross hematuria for 6 months and elevated serum creatinine for 1 month. She was in good health until November of 2009, when she noticed gross hematuria without fever, dysuria, frequency, urgency, and suprapubic pain. Hematuria was noted every month, but she did not pay it any attention. In March of 2010, she noticed edema of her lower extremities and went to a local hospital. Laboratory analysis showed urine protein 7.28 g/24 h, RBC 823 per high-power field, Hemoglobin 8.5 g/dl, serum albumin 3.1 g/dL, creatinine 4.98 mg/dL. One month later, she was transferred to our hospital. On physical examination, she presented with a pale face and edema of the lower extremities. Blood pressure was 150/100 mmHg, Hemoglobin was 7.4 g/dL, while the leukocyte and platelet counts were normal. Serum creatinine was 7.15 mg/dL and albumin 2.8 g/dL. Urinalysis showed hematuria (484 RBCs per high-power field) and proteinuria 4+. Antinuclear antibody, complement levels and ANCAs were all negative or normal. Renal ultrasound showed normal-sized kidneys without obstruction or masses. Serum anti-GBM antibody assay by enzyme immunoassays (ELISA, EA 1251-9601 G, EUROIMMUN Medical Laboratory Diagnostics Co.,Ltd) showed 119.70 RU/mL (normal range, <20 RU/mL). She became weaker and serum creatinine gradually elevated to 9.3 mg/dL while urine output decreased to 300 ml/day. No hemoptysis developed and chest X-ray was normal.

**Figure 1:**
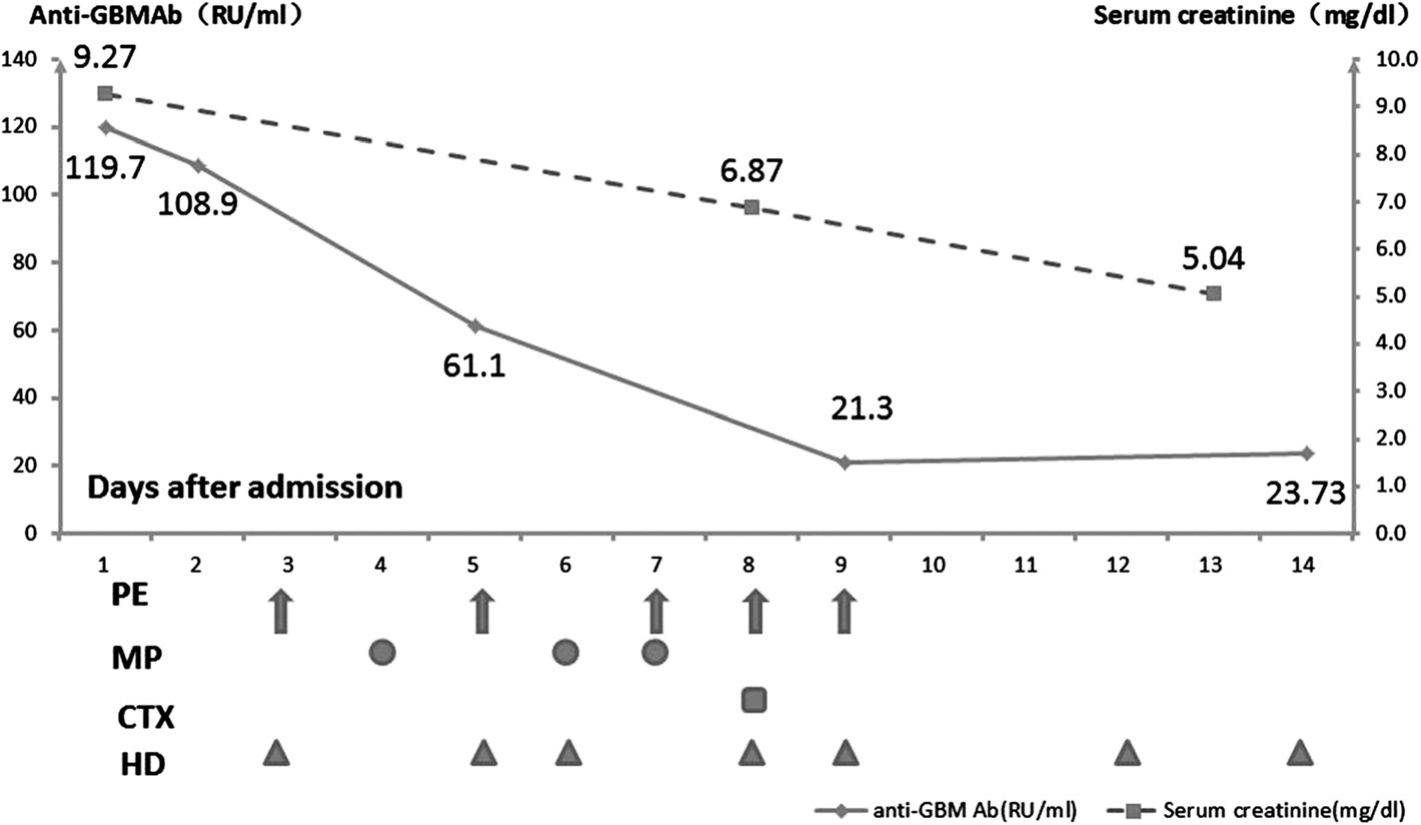
**Changes of serum creatinine, anti-GBM antibody and therapy in the patient with anti-GBM nephritis.** Left Y-axis: rhombus: solid line: serum anti-GBM antibody (RU/mL); Right Y-axis: squares: dashed line: serum creatinine concentration (mg/dL). PE: plasma exchange, MP: methylprednisolone, CTX: cyclophosphamide, HD: hemodialysis.

The patient was diagnosed with anti-GBM nephritis, then treated with plasma exchange (2000-3000 ml plasma/exchange) plus hemodialysis every other day for 5 times, methylprednisolone 0.5 g for three days, plus cyclophosphamide 1 g for pulse infusion. Then her medications were shifted to oral prednisone 50 mg/day. 2 weeks later, MP and CTX were administered again. Although serum anti-GBM antibody titer decreased gradually to normal, serum creatinine and urine outcome did not improve (Figure 1). Hemodialysis therapy continued.

Surprisingly, her identical twin sister came to our hospital complaining of malar erythema and arthralgia one month later. No hemoptysis, photosensitivity, edema and hematuria were recorded. Physical examination showed butterfly rash. No edema, oral ulcer, fever and hair loss. Hemoglobin was 13.1 g/dL, WBC was 5.46x10^9^/L, platelet was 116x10^9^/L, serum albumin was 4.17 g/dL and creatinine was 0.56 mg/dL. Urine protein was 0.1 g/24 h, No microscopic hematuria was revealed. Antinuclear antibody was positive with liter 1:1000, Smith (Sm) antibody ++, dsDNA antibody negative, ANCA negative. Anti-GBM antibody and complements were within their normal ranges (C3 1.07 g/L, C4 0.139 g/L). Based on the diagnostic criteria of SLE in 1982 of American College of Rheumatology, she was diagnosed as SLE. Oral prednisone 40 mg/day was initiated and tapered regularly with alleviation of her rash and arthralgia. Two years later, malar erythema relapsed with lowered C3 0.652 g/L. Prednisone was increased to 40 mg/day combined with leflunomide 20 mg/day and hydroxychloroquine 400 mg/day. Her rash faded again. At the end of follow up, serum creatinine was 0.71 mg/dL, serum albumin was 4.18 g/dL, hemoglobin was 14.2 g/dL, WBC was 4.25x10^9^/L, platelet was 114x10^9^/L, anti-GBM antibody was still within normal range.

Considering HLA-DRB1*1501 alleles are associated with susceptibility to anti-GBM nephritis and lupus, we analyzed HLA-DRB1 alleles in these two sisters, and they were both HLA-DRB1*1501 homozygotes.

## Conclusions

Anti-GBM nephritis is a rare autoimmune disorder characterized by elevated serum anti-GBM antibodies and rapidly progressive glomerulonephritis. Renal biopsy reveals crescent glomerulonephritis with linear IgG deposit along capillary walls. When pulmonary hemorrhage is also present, this condition is called Goodpasture’s Syndrome [3]. The first patient presented gross hematuria and rapid deterioration of renal function with significantly elevated anti-GBM antibodies and negative ANCA and ANA. Although renal biopsy was not conducted, anti-GBM nephritis was diagnosed based on elevated anti-GBM antibody and clinical manifestations of rapidly progressive glomerulonephritis. Although plasma exchange and pulse treatment of MP and CTX had been applied, she was still dialysis-dependent. The outcomes of anti-GBM nephritis depend on renal function at presentation and percentage of crescents on renal biopsy [4]. Considering she was dialysis-dependent and serum creatinine exceeded 9 mg/dL at presentation, chance of renal survival should be less than 8% [4]. Interestingly,her identical twin sister developed lupus with negative anti-GBM antibody later. Till now, it has not been reported that identical twins developed either lupus or anti-GBM nephritis.

There are evidently differences in pathogenesis, clinical manifestations and outcomes between anti-GBM glomerulonephritis and lupus. For example, anti-GBM nephritis is associated with anti-GBM antibody and rarely relapses, while lupus involves many antibodies including ANA, dsDNA, Smith(Sm) antibody, and is a life-long disorder that may relapse. It has long been believed that anti-GBM nephritis is different from other autoimmune disorders.

Recently, there is evidence suggesting that there is relationship between lupus and anti-GBM nephritis. C-H Li et al. found that anti-GBM antibody was detected in 14 (8.9%) of 157 patients with SLE [5]. All of the 14 patients developed lupus nephritis; ten of them reached the criteria for crescentic glomerulonephritis and five of them were diagnosed as Goodpasture’s disease. A few cases also showed coexistence of anti-GBM glomerulonephritis and lupus [6,7]. It was suggested that ongoing glomerular inflammation may change GBM structure, triggering an autoimmune reaction by exposing or modifying antigens, as proposed for the association between anti-GBM disease and ANCA glomerulonephritis [8]. However, identification of twins diagnosed as anti-GBM glomerunephritis and lupus respectively could not be attributed to this hypothesis. It seems that some genetic connection may exist between these two diseases.

Human leukocyte antigen (HLA), which plays an important role in immune responses, consists of the class I and class II molecules. Class II molecules are required for the presentation of antigens to T cell receptors and contribute to the production of specific antibodies. Genetic defects in self-tolerance can be triggered by reduced or altered expression of HLA molecules, which may aggravate the progression of different antoimmune diseases [9]. Genes in the HLA complex are among the strongest predisposing genetic factors. HLA-DRB1*1501 has been associated with susceptibility to anti-GBM disease. The frequency of HLA-DRB1*1501 in anti-GBM antibody-positive Japanese and Chinese patients was significantly higher than controls (36/88 vs 64/400, P = 1.597 × 10^−7^, in China) [10,11]. HLA-DRB1*1501 alleles were also significant risk factors for SLE [9,12]. DRB1*1501/DQB1*0602 was identified as one of three microsatellite-inferred risk haplotypes in European lupus families [13]. HLA-DRB1*1501 allele are strongly associated with multiple sclerosis in African-American [14]. The mechanism underlying HLA association with autoimmune diseases is not clearly understood until now. Although it is difficult to define HLA-DRB1*1501 as a molecular biomarker of anti-GBM nephritis or lupus, the fact that these identical twins were both HLA-DRB1*1501 homozygotes supports the possible association of this allele with anti-GBM and lupus.

The latest research shows that out of about 25 different molecules in anti-GBM nephritis mouse model and spontaneous lupus nephritis (SLN), all influenced both diseases concordantly, including complement-and FcR-dependent activation of resident renal cells and infiltrating leukocytes, proinflammatory mediators initially, and profibrotic molecules [15]. Since SLN in mouse models takes 6-12 months to manifest, the experimental anti-GBM nephritis model has been used as a useful tool to unravel the molecular basis of SLN.

It is exciting that kallikrein genes may be the common candidate genes for lupus and anti-GBM nephritis in animal models [16]. Briefly, some murine strains, including *NZW*, *DBA/1*, are highly sensitive to experimental anti-GBM nephritis, while other strains such as C57BL/6(B6) are resistant [17]. A microarray-based transcriptomic analysis revealed that the kallikrein (*KLK*) gene family may be the key to produce these differences between these strains [16]. The tissue kallikrein gene cluster is located within the *Sle3* interval [18], one of potential pathogenic loci in SLE. B6. Sle3^*z*^ mice (bearing the NZM2410/NZM-derived “z” allele of Sle3 on the relatively normal B6 background), exhibited increased susceptibility to experimental anti-GBM-induced nephritis compared with wide-type B6 [16]. Delivery of *klk1* encoding KLK1(the principal kinin-generating enzyme) to B6. Sle3 congenics ameliorated anti-GBM-induced nephritis [19]. Interestingly, B6. NZMc1|c7 mice (bicongenic for *Sle1* and *Sle3*) could produce significantly elevated glomerular-binding autoantibodies compared with B6 [20]. These findings indicated that kallikreins play an important role in these diseases, and could constitute a potential candidate genes for anti-GBM nephritis and SLN.

Although evidence mentioned above demonstrates similarities of genetic backgrounds between lupus and anti-GBM nephritis, after all, the huge differences of their clinical features still exist. Why would these twins bearing identical genetic backgrounds develop such different diseases respectively? Although genetic predisposition plays an important role in these diseases, it is generally accepted that environmental factors modulate the susceptibility, in part, through epigenetic changes. The concordance rate of lupus in identical twins is greater than in the general population, but it is still incomplete [1,21,22]. Many epidemiologic and environmental elements such as ethnicity, gender, hormonal exposure, UV radiation, pregnancy, smoking habits, and viral exposures are thought to influence these phenotypic variations. Recent studies showed that epigenetic alterations play an important role on the pathogenesis of SLE, including the global loss of DNA methylation and altered patterns of histone modifications [23]. Although anti-GBM glomerulonephritis may occur in siblings and sets of identical twins [2], there are few epigenetic study of this disease because of the rarity. Environmental factors including exposure to hydrocarbons and smoking have been thought to associate with the onset and progression of anti-GBM glomerulonephritis, which is different from SLE. Because pathogenic mechanisms involving the onset and progression of these diseases are far from clear, although the twins seemed to grow up in the same environment and shared the same genetic background, it is possible that some unknown environmental factors triggered the onsets of diverse diseases. Although the girl suffering from SLE has normal serum anti-GBM antibody, she may also develop anti-GBM glomerulonephritis in the future under some situation, and vice versa.

We present two identical twins that developed anti-GBM nephritis and lupus respectively, which supports a connection between these diseases, which need to be investigated further. Environmental factors may contribute to final clinical differences.

## Consent

Written informed consent was obtained from both patients for publication of this Case report and any accompanying images. A copy of the written content is available for review by the Editor of this journal.

## Abbreviations

SLE: Systemic lupus erythematosus; GBM: Glomerular basement membrane; HLA: Human leukocyte antigen; ANCA: Anti-neutrophil cytoplasmic antibody; MP: Methylprednisolone; CTX: Cyclophosphamide.

## Competing interests

None of the authors has any competing interests.

## Authors’ contributions

XXL, YW, YT, PF and ZXH were the physicians who treated the patient in this report. YY and JW performed genetic studies. The manuscript was prepared by XXL, YW, YY, JW, YT, PF and ZXH. All authors participated in discussions about the manuscript and approved the final version.

## Pre-publication history

The pre-publication history for this paper can be accessed here:

http://www.biomedcentral.com/1471-2369/14/277/prepub
